# 液相色谱-四极杆/静电场轨道阱质谱法测定牛奶中22种真菌毒素

**DOI:** 10.3724/SP.J.1123.2023.07010

**Published:** 2023-11-08

**Authors:** Lanyan TONG, Bozhou XU, Xuemei NIE, Xiujuan WANG, Jiahui MA, Wei GUO, Genrong LI, Yingkun GONG, Xiuli XU

**Affiliations:** 1.中国检验检疫科学研究院, 北京 100176; 1. Chinese Academy of Inspection and Quarantine, Beijing 100176, China; 2.国家市场监管重点实验室(食品质量与安全), 国家乳业技术创新中心, 北京 100176; 2. Key Laboratory of Food Quality and Safety for State Market Regulation, National Center of Technology Innovation for Dairy, Beijing 100176, China; 3.重庆市计量质量检测研究院, 国家农副加工产品及调味品质量检验检测中心, 重庆 401123; 3. Chongqing Academy of Metrology and Quality Inspection, National Center of Quality Supervision & Inspection Agricultural Processed Products and Condiments, Chongqing 401123, China

**Keywords:** 液相色谱-四极杆/静电场轨道阱质谱, 快速前处理, 真菌毒素, 牛奶, liquid chromatography-quadrupole/orbitrap mass spectrometry, rapid pretreatment, mycotoxins, milk

## Abstract

真菌毒素因具有致癌性、致突变性、肝毒性、肾毒性、免疫毒性、神经毒性及致畸性等危害而广受关注。本研究建立了高效液相色谱-四极杆/静电场轨道阱质谱法同时测定牛奶中22种真菌毒素的分析方法。针对真菌毒素的特性和牛奶样品基质的特点选择简单、有效、快速的前处理方法,重点优化了提取溶剂种类、溶剂用量和提取盐用量。样品用0.5%甲酸乙腈提取,氯化钠分层,低温高速离心净化,上清液经氮吹浓缩后复溶,ZORBAX Eclipse Plus C_18_色谱柱(100 mm×2.1 mm, 1.7 μm)分离,同位素内标法定量。化合物在正离子模式下电离,在数据依赖型质谱扫描模式下对目标物进行定性。结果表明,22种目标物在0.5~100.0 μg/L线性范围内呈良好的线性关系,相关系数(*R*^2^)均大于0.999,该方法的检出限(*S/N*=3)为0.3~0.5 μg/kg,定量限(*S/N*=10)为1.0~1.5 μg/kg。以牛奶为基质,在1.5、5.0、15 μg/kg加标水平下,22种真菌毒素的回收率为84.7%~100.8%,相对标准偏差为1.2%~9.9%。应用本方法对25份实际牛奶样品进行分析检测,结果显示22种真菌毒素均未检出。该方法灵敏度好,准确度高,可作为牛奶中真菌毒素的高通量筛查和确证检测方法。

## Abstract (en)

Mycotoxins have carcinogenic, mutagenic, hepatotoxic, nephrotoxic, immunotoxic, neurotoxic, and teratogenic properties. Thus, these substances have attracted significant attention because they pose a threat to human health. As research on mycotoxins deepens, new structural analogues of mycotoxins are constantly being discovered. In this study, a method based on high performance liquid chromatography-quadrupole/orbitrap mass spectrometry was established for the simultaneous determination of 22 mycotoxins in milk. A simple, effective, and rapid pretreatment method was optimized by focusing on the solvent type, extractant volume, and extracting salt based on the characteristics of the mycotoxins and sample matrix. The analytes were extracted using 0.5% formic acid acetonitrile solution and added with sodium chloride to separate fats from water. The samples were centrifuged at 8000 r/min (4 ℃) for 5 min using a centrifuge and then concentrated using nitrogen. The dry residue was dissolved with 50% methanol aqueous solution. Twenty-two mycotoxins were separated on a ZORBAX Eclipse Plus C_18_ chromatographic column (100 mm×2.1 mm, 1.7 μm), and quantitative analysis was performed using the isotope internal standard method. The analytes were determined by liquid chromatography-quadrupole/orbitrap mass spectrometry in positive electrospray ionization mode. Qualitative analyses of the compounds were performed in full mass spectrometry/data-dependent tandem mass spectrometry (MS/dd-MS^2^) mode. Good linearities in the range of 0.5-100.0 μg/L were observed for the 22 mycotoxins, and the correlation coefficients (*R*^2^) were greater than 0.999. The limits of detection (*S/N*=3) and quantification (*S/N*=10) ranged from 0.3 to 0.5 μg/kg and from 1.0 to 1.5 μg/kg, respectively. The average recoveries of the 22 mycotoxins at three spiked levels of 1.5, 5.0, and 15 μg/kg were between 84.7% and 100.8%, with relative standard deviations (RSDs) of 1.2%-9.9%. These findings indicate that the method has high sensitivity and accuracy as well as good precision. Finally, the method was applied to the detection and analysis of mycotoxins in 25 actual commercial milk samples. The results revealed that the selected samples were not contaminated with any of the mycotoxins analyzed. Thus, the proposed method is useful as a quick preprocessing and confirmatory method for the simultaneous determination of mycotoxins in milk.

自“三聚氰胺”事件发生以来,乳品的安全问题引起了广大消费者及相关监管部门的关注^[1,2]^。近年来,关于乳及乳制品中检测出真菌毒素类物质的事件时有报道^[3⇓-5]^。真菌毒素是真菌在粮食和饲料中所产生的代谢产物,毒素通过食物链从牛、羊等转移至乳及乳制品中,受污染的乳及乳制品即使经过高温、杀菌过程,真菌毒素也不会被破坏^[6]^。真菌毒素是由镰刀菌属、青霉属、曲霉属等真菌在适宜的温、湿度条件下产生的次级代谢产物^[7]^。大多数真菌毒素可通过抑制动物体内蛋白质和相关酶的合成破坏细胞结构,导致动物体肝脏^[8]^、肾脏^[9]^、神经^[10]^、造血^[11]^等组织器官损害,具有致癌、致畸、致突变、神经毒性以及免疫毒性等作用,严重威胁人类身体健康。随着对真菌毒素研究的深入,一些新型的真菌毒素不断被发现,如单端孢霉烯族毒素、恩镰孢菌毒素、白僵菌素和细胞松弛素等^[12]^。这些真菌毒素尚未得到监管,没有被标准检测方法覆盖,且毒理研究和污染数据有限,还能与其他真菌毒素协同作用产生更强毒性,因此被称为“新兴”真菌毒素。目前国内对于乳中黄曲霉毒素M_1_(AFM_1_)的限量标准为0.5 μg/kg,其他真菌毒素均未单独制定残留限量标准。且现行GB 5009.22-2016^[13]^仅适用于乳中黄曲霉毒素M_1_和M_2_(AFM_2_)的测定。因此,针对尚未制定国家标准限量或者研究较少的真菌毒素亟待开展相关研究。

文献报道的真菌毒素检测方法主要有免疫分析法^[14,15]^、色谱法^[16,17]^和质谱法^[18,19]^。质谱法简化了前处理步骤,提高了准确性和灵敏度,目前广泛应用于真菌毒素的检测。三重四极杆质谱定量准确,但是分辨率不足。液态乳中脂肪含量高,基质复杂,且真菌毒素含量较低,实现多种真菌毒素的高通量筛查及同时检测存在极大挑战。液相色谱-四极杆/静电场轨道阱质谱法具有质量分辨率高和提供准确相对分子质量的优势,无需逐个优化目标化合物的质谱参数,不受传统三重四极杆质谱检测化合物数量的限制,可通过提供母离子和碎片离子的精确质量数,实现复杂基质中目标物的准确定性^[20,21]^。因此本研究根据液态牛奶的基质特点,采用液相色谱-四极杆/静电场轨道阱质谱分析系统,建立了牛奶中22种真菌毒素同时测定的分析方法,通过一级全扫描获得目标物的精确质量数,同时结合数据依赖型二级扫描(dd-MS^2^)模式获得目标物的二级特征质谱图,快速、准确、高效地完成了牛奶中22种真菌毒素的快速筛查和确证,以期为真菌毒素污染风险全面评估及防控技术研发提供思路。

## 1 实验部分

### 1.1 仪器与试剂

Dionex Ultimate 3000超高效液相色谱仪(美国Dionex公司); Thermo Q-Exactive Orbitrap四极杆/静电场轨道阱质谱仪(美国Thermo Fisher公司);电子天平XP 105DR(瑞士Mettler Toledo公司);移液器(德国Eppendorf公司); Vortex-Genie2多功能旋涡混合器(美国Scientific Industries公司); KQ-500DE数控超声波清洗器(昆山市超声仪器有限公司); Allegra X-22R离心机(美国贝克曼库尔特有限公司); MFV-24智能氮吹仪(得泰仪器科技有限公司)。

22种真菌毒素标准品:恩镰孢菌素A(ENA, CAS号:2503-13-1,纯度≥99.0%)、恩镰孢菌素A_1_(ENA_1_, CAS号:4530-21-6,纯度≥99.9%)、恩镰孢菌素B(ENB, CAS号:917-13-5,纯度≥99.0%)、恩镰孢菌素B_1_(ENB_1_, CAS号:19914-20-6,纯度≥99.5%)、白僵菌素(BEA, CAS号:26048-05-5,纯度≥99.2%)、细胞松弛素B(CB, CAS号:14930-96-2,纯度≥99.0%)、细胞松弛素C(CC, CAS号:22144-76-9,纯度≥99.2%)、细胞松弛素D(CD, CAS号:22144-77-0,纯度≥98.5%)、细胞松弛素E(CE, CAS号:36011-19-5,纯度≥99.2%)、细胞松弛素H(CH, CAS号:53760-19-3,纯度≥99.2%)、细胞松弛素J(CJ, CAS号:53760-20-6,纯度≥99.5%)、二乙酰镰刀菌烯醇(CAS号:2270-40-8, DAS,纯度≥99.0%)、新茄病镰刀菌烯醇(NEO, CAS号:36519-25-2,纯度≥99.3%)、T-2毒素(T-2, CAS号:21259-20-1,纯度≥98.2%)、HT-2毒素(HT-2, CAS号:26934-87-2,纯度≥99.0%)、黄曲霉毒素B_1_(AFB_1_, CAS号:1162-65-8,纯度≥99.0%)、黄曲霉毒素B_2_ (AFB_2_, CAS号:7220-81-7,纯度≥99.9%)、黄曲霉毒素M_1_(CAS号:6795-23-9,纯度≥99.9%)、黄曲霉毒素M_2_(CAS号:6885-57-0,纯度≥99.2%)、赭曲霉毒素A(OTA, CAS号:303-47-9,纯度≥99.0%)、赭曲霉毒素B(OTB, CAS号:4825-86-9,纯度≥99.0%)、赭曲霉毒素C(OTC, CAS号:4865-85-4,纯度≥99.0%)购自澳大利亚Romer公司,纯度均在97%以上。3种同位素内标:^13^C_17_-黄曲霉毒素B_1_(^13^C_17_-AFB_1_, CAS号:1217449-45-0,纯度≥99.5%)、^13^C_20_-赭曲霉毒素A(^13^C_20_-OTA, CAS号:911392-42-2,纯度≥99.5%)、^13^C_15_-瓜萎镰菌醇(^13^C_15_-NIV, CAS号:911392-40-0,纯度≥99.5%),购于美国Romer Labs公司。

甲醇、乙腈、甲酸、乙酸(色谱纯,美国Thermo Fisher公司);乙酸铵(色谱纯,德国西格玛奥德里奇贸易有限公司);超纯水(由Milli-Q超纯水机制备(英国ELGA公司));氯化钠(分析纯,美国Agilent公司);尼龙滤膜(0.22 μm,美国Waters公司)。

### 1.2 标准溶液的配制

标准储备液:向真菌毒素标准品中加入适量甲醇,充分溶解得到真菌毒素标准储备液,于-20 ℃冰箱避光封口保存,12个月内用完。标准工作液:分别精密移取适量22种真菌毒素标准储备液于10 mL容量瓶中,用甲醇稀释定容,配制成质量浓度为1 mg/L的混合标准工作液,涡旋混匀后置于棕色试剂瓶内,于-20 ℃冰箱避光封口保存。根据实验需要,用甲醇配制成不同浓度的混合标准工作液,现用现配。

内标储备液:向同位素内标标准品中加入适量甲醇,充分溶解得到内标储备液,于-20 ℃冰箱避光封口保存,12个月内用完。内标使用液:分别精密移取适量3种同位素内标储备液于容量瓶中,用甲醇稀释定容至刻度,配成质量浓度为200 μg/L的同位素内标使用液,于-20 ℃冰箱避光封口保存,3个月内用完。

### 1.3 样品前处理

称取均匀试样2.0 g,置于15 mL离心管中,加入10 μL内标使用液和4 mL 0.5%甲酸乙腈溶液,涡旋1 min,超声提取10 min,加入1 g氯化钠,涡旋2 min,在4 ℃条件下10000 r/min离心10 min,准确移取2 mL上清液于氮吹管中,于40 ℃水浴中氮吹至干,加入1 mL 50%甲醇水溶液复溶,涡旋1 min,过0.22 μm滤膜,供液相色谱-四极杆/静电场轨道阱质谱系统分析。

### 1.4 分析条件

#### 1.4.1 色谱条件

色谱柱:ZORBAX Eclipse Plus C_18_色谱柱(100 mm×2.1 mm, 1.7 μm,美国Agilent公司);柱温:35 ℃;流动相A为含10 mmol/L乙酸铵的0.1%乙酸水溶液,流动相B为0.1%乙酸甲醇溶液;流速0.30 mL/min;进样量5.0 μL。梯度洗脱条件如表1。

**表 1:** 梯度洗脱程序

Time/min	φ(A)/%	φ(B)/%
0	90	10
2.0	90	10
3.0	80	20
7.0	76	24
10.5	70	30
13.5	40	60
15.0	30	70
18.0	25	75
18.1	0	100
21.0	0	100
21.1	90	10
22.0	90	10

A: 0.1% acetic acid aqueous solution containing 10 mmol/L ammonium acetate; B: 0.1% acetic acid methanol solution.

#### 1.4.2 质谱条件

电喷雾离子源(ESI),正离子模式;离子源温度320 ℃;鞘气流速40 arb;辅助气流速10 arb;喷雾电压3.2 kV,毛细管温度320 ℃。扫描模式为数据依赖型二级扫描模式。全扫描模式的分辨率为70000半峰全宽(FWHM),扫描范围为*m/z* 100~1000, C-trap的自动增益控制(AGC)目标为1×10^6^,最大注射时间(IT)为100 ms。dd-MS^2^的分辨率为17500 FWHM, AGC目标1×10^5^,最大IT 50 ms, TopN 5,隔离窗口*m/z* 4。22种真菌毒素及3种同位素内标的质谱信息见表2。

**表 2:** 22种真菌毒素及3种同位素内标的化合物信息及质谱参数

No.	Compound	t_R_/min	Adduct	Product ions (m/z)	Collision energy/eV	IS
1	enniatin A (ENA)	19.96	[M+NH_4_]^+^	210.14893^*^, 228.15898, 100.11282	35	^13^C_15_-NIV
2	enniatin A_1_(ENA_1_)	19.92	[M+NH_4_]^+^	210.14923^*^, 228.15998, 100.11278	35	^13^C_15_-NIV
3	enniatin B (ENB)	19.80	[M+NH_4_]^+^	228.15892^*^, 210.14891, 214.14413	30	^13^C_15_-NIV
4	enniatin B_1_(ENB_1_)	19.85	[M+NH_4_]^+^	228.15938^*^, 214.14395, 210.14891	30	^13^C_15_-NIV
5	beauveria (BEA)	19.86	[M+NH_4_]^+^	228.23222^*^, 214.21654, 262.14380	25	^13^C_15_-NIV
6	cytochalasin B (CB)	16.04	[M+H]^+^	264.13800^*^, 252.13766, 120.08121	35	^13^C_15_-NIV
7	cytochalasin C (CC)	16.04	[M+NH_4_]^+^	265.15900^*^, 237.16402, 145.10091	40	^13^C_15_-NIV
8	cytochalasin D (CD)	16.65	[M+NH_4_]^+^	265.15918^*^, 237.16409, 120.08099	40	^13^C_15_-NIV
9	cytochalasin E (CE)	16.92	[M+NH_4_]^+^	258.14920^*^, 120.08103, 159.08026	30	^13^C_15_-NIV
10	cytochalasin H (CH)	16.14	[M+Na]^+^	269.19000^*^, 120.08096, 91.05484	45	^13^C_15_-NIV
11	cytochalasin J (CJ)	15.46	[M+H]^+^	251.17976^*^, 120.08119, 91.05489	35	^13^C_15_-NIV
12	diacetoxyscirpenol (DAS)	14.73	[M+NH_4_]^+^	247.13100^*^, 229.12137, 217.11879	30	^13^C_15_-NIV
13	neosolaniol (NEO)	8.85	[M+NH_4_]^+^	245.11765^*^, 227.10090, 215.10712	30	^13^C_15_-NIV
14	HT-2 toxin (HT-2)	15.71	[M+NH_4_]^+^	245.11763^*^, 227.10698, 215.10699	30	^13^C_15_-NIV
15	T-2 toxin (T-2)	16.38	[M+NH_4_]^+^	245.11766^*^, 227.10707, 215.10712	25	^13^C_15_-NIV
16	aflatoxin B_1_(AFB_1_)	14.67	[M+H]^+^	285.07553^*^, 298.04498, 270.05136	40	^13^C_17_-AFB_1_
17	aflatoxin B_2_(AFB_2_)	14.38	[M+H]^+^	285.09134^*^, 287.09088, 259.05988	40	^13^C_17_-AFB_1_
18	aflatoxin M_1_(AFM_1_)	14.02	[M+H]^+^	301.06983^*^, 273.07586, 229.04294	45	^13^C_17_-AFB_1_
19	aflatoxin M_2_(AFM_2_)	13.65	[M+H]^+^	313.06808^*^, 285.07479, 273.08040	45	^13^C_17_-AFB_1_
20	ochratoxin A (OTA)	16.75	[M+H]^+^	271.03467^*^, 257.01953, 385.08221	40	^13^C_20_-OTA
21	ochratoxin B (OTB)	15.86	[M+H]^+^	237.07414^*^, 223.05847, 324.12140	40	^13^C_20_-OTA
22	ochratoxin C (OTC)	19.27	[M+H]^+^	271.03491^*^, 358.08127, 257.01932	40	^13^C_20_-OTA
23	^13^C_17_-aflatoxin B_1_(^13^C_17_-AFB_1_)	14.57	[M+H]^+^	301.12848^*^, 285.10211, 272.13034	35	
24	^13^C_20_-ochratoxin A (^13^C_20_-OTA)	16.74	[M+H]^+^	268.05746^*^, 250.04707, 360.12003	35	
25	^13^C_15_-nivalenol (^13^C_15_-NIV)	19.80	[M+3H]^+^	107.04939^*^, 163.11142, 201.09058	35	

* Quantitative ion.

### 1.5 数据分析

实验分别采用SPSS 22.0和Origin 2018软件进行统计分析和绘图。

## 2 结果与讨论

### 2.1 质谱条件优化

质谱条件是影响真菌毒素前体离子和碎片离子响应强度的关键因素。本实验优化了电离模式和归一化碰撞能。利用液相色谱-四极杆/静电场轨道阱质谱对22种真菌毒素混合标准溶液进行正负离子扫描,得到一级全扫描质谱图,发现部分真菌毒素在负离子模式下无响应,22种真菌毒素在正离子模式下响应值均高于负离子模式,最终确定扫描模式为正离子模式。为了获得最大响应和稳定的碎片离子,比较了真菌毒素在不同碰撞能量下的碎片离子强度。22种真菌毒素和3种内标物的保留时间和质谱参数见表2。

### 2.2 色谱条件优化

#### 2.2.1 色谱柱的选择

色谱柱的类型、填料和长度是影响目标化合物分离的重要因素之一。选择合适的色谱柱,目标物可以得到更好的分离,也可以提高方法的灵敏度和稳定性。本文比较了ZORBAX Eclipse Plus C_18_ (100 mm×2.1 mm, 1.7 μm)、Waters BEH HILIC (100 mm×2.1 mm, 1. 7 μm)和SUPELCO Ascentis C_8_ (100 mm×4. 6 mm, 1.7 μm)3种色谱柱的分离效果。结果显示,Waters BEH HILIC和SUPELCO Ascentis C_8_色谱柱无法对细胞松弛素C和细胞松弛素D这对同分异构体进行有效分离,而ZORBAX Eclipse Plus C_18_色谱柱则可以对这对同分异构体进行分离,因此采用该色谱柱分析牛奶中的22种真菌毒素。

#### 2.2.2 流动相的选择

流动相、pH值、流速等都会影响目标化合物的出峰时间和响应强度。本实验考察了甲醇和乙腈作为强洗脱流动相的洗脱效果。结果表明,甲醇-水作为流动相时,化合物具有更好的响应。这有可能是因为甲醇具有质子供体特性,有利于真菌毒素形成带正电荷的分子离子加合物。乙腈的介电常数低于甲醇,因此甲醇的电荷转移高于乙腈,电离效率更高。

由于酸性环境有利于[M+H]^+^的形成,有利于提高离子化效率^[22,23]^,实验在流动相中加入0.1%乙酸,发现使用0.1%乙酸水和0.1%乙酸甲醇进行梯度洗脱时,黄曲霉毒素类化合物有拖尾现象,而在水相中加入少量乙酸铵能消除这种拖尾现象。为了增大目标化合物的响应值并改善峰形,实验进一步考察了不同浓度乙酸铵对目标化合物离子化效率的影响。结果表明,10 mmol/L的乙酸铵能够最大限度地增加目标化合物的响应值并改善峰形,而过高浓度的乙酸铵反而会降低目标化合物的离子化效率^[24]^。所以实验最终选择含10 mmol/L乙酸铵的0.1%乙酸水溶液和0.1%乙酸甲醇溶液作为流动相。22种真菌毒素和3种同位素内标在最优实验条件下的色谱图如图1所示。

**图1:**
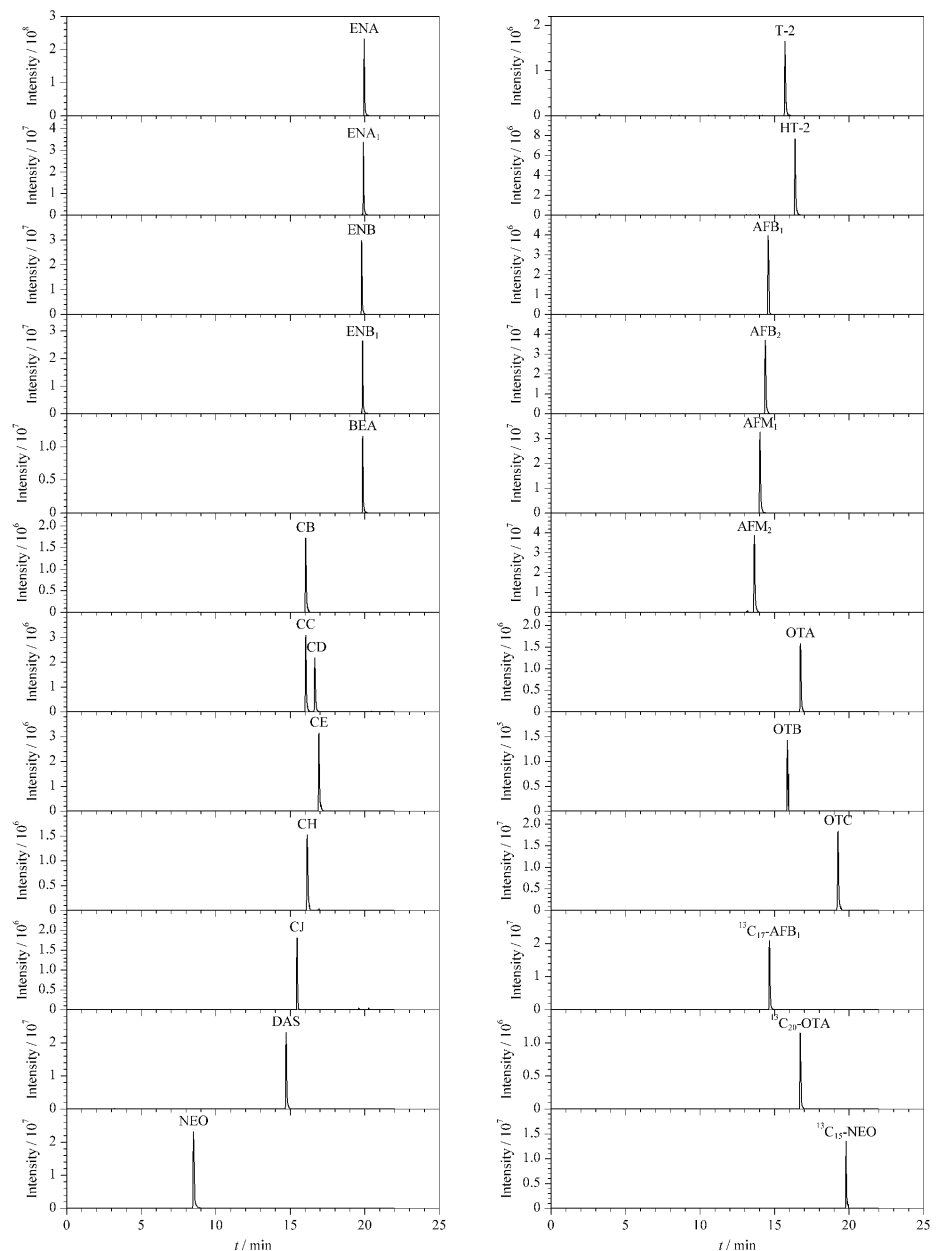
22种真菌毒素和3种同位素内标的提取离子流色谱图

### 2.3 前处理方法的优化

#### 2.3.1 提取溶剂的选择

根据文献[21,25]得知,提取真菌毒素常用的提取溶剂为甲醇、乙腈、甲醇-水和乙腈-水。由于牛奶中有较高含量的蛋白质和脂肪等,在真菌毒素的提取过程中容易团聚和乳化而影响提取效果,因此需要对牛奶进行去除蛋白质和脂肪的操作,以提高提取效率。本实验比较了甲醇、乙腈、甲醇-乙腈(1∶1, v/v)对真菌毒素提取效果的影响。结果显示,乙腈具有更好的提取效果,因为乙腈有很好的沉淀蛋白质的作用。在提取溶剂中加入一定量的酸性物质可以改善真菌毒素的提取效率,本实验比较了不同体积分数(0、0.5%、1.0%、2.0%、4.0%和6.0%)的甲酸乙腈溶液的提取效率,实验结果如图2所示。在加入酸性物质后,真菌毒素的提取效果得到改善,但是过高的酸性比例会降低提取效率,综合考虑确定0.5%甲酸乙腈作为提取溶剂。

**图2:**
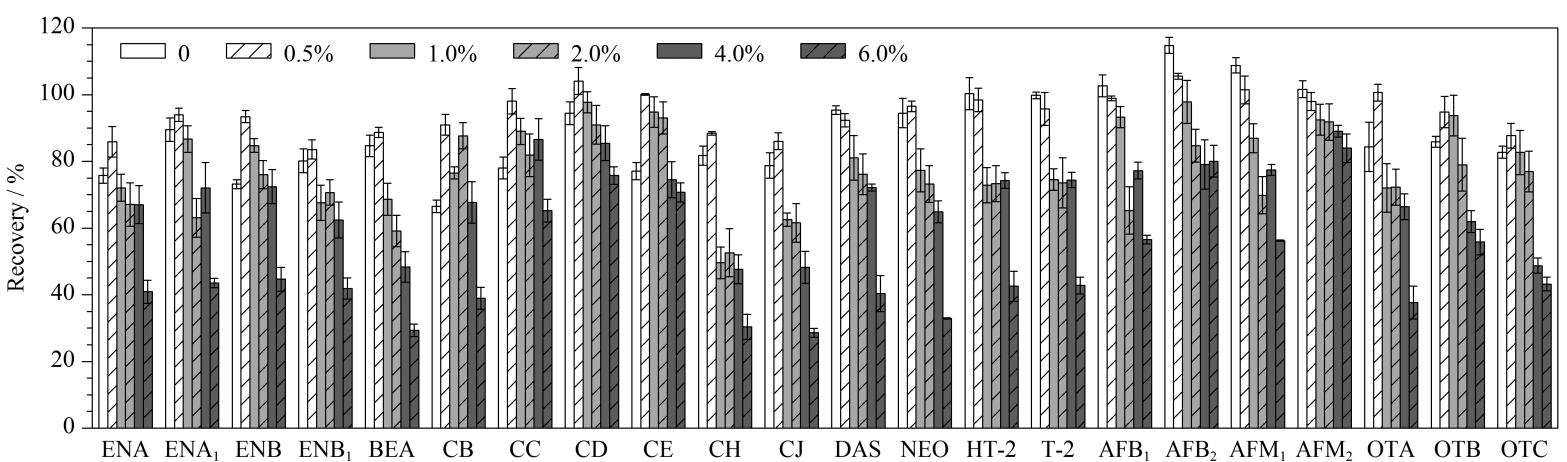
采用含不同体积分数甲酸的乙腈提取时22种真菌毒素的回收率(*n*=6)

#### 2.3.2 提取溶剂体积的选择

本实验对比了提取溶剂体积为2、4、8 mL时22种真菌毒素的回收率情况(见图3)。大部分真菌毒素回收率随着提取溶剂体积的增加而提高,但回收率不会随着提取溶剂用量增加持续提高。部分目标物如ENB、OTA、OTB、OTC随着提取溶剂体积的增加回收率呈先增加后减小的趋势,这可能由于提取溶剂体积增加,提取到的杂质也随之增加,干扰目标物检测。而且实验发现进一步提高溶剂使用量,氯化钠盐析和高速离心无法实现水相和有机相分层,不利于目标化合物的析出。因此,最终提取溶剂体积选择4 mL。

**图3:**
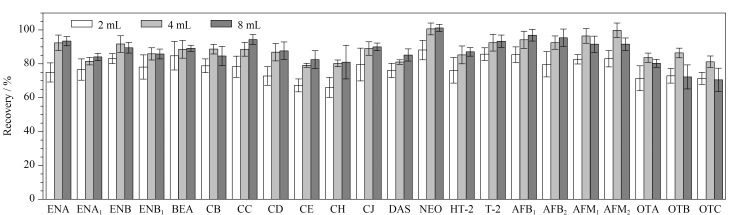
不同提取溶剂体积下22种真菌毒素的回收率(*n*=6)

#### 2.3.3 提取盐的选择

牛奶中真菌毒素的提取属于液液萃取,加入一定量的盐对于真菌毒素提取有十分重要的作用。本文考察了氯化钠和无水硫酸镁对回收率的影响,由图4可知,在牛奶提取过程中加入氯化钠,22种真菌毒素的回收率较好。氯化钠在实验过程中的盐析作用有利于真菌毒素从牛奶中析出,离心过后有机相和水相分层,目标化合物进入有机相,故加入氯化钠有利于提高回收率。在含水的牛奶样品中加入无水硫酸镁,无水硫酸镁吸水后放热,牛奶蛋白质变质结块,吸附部分目标化合物,导致部分目标化合物回收率降低。故本实验选择加入氯化钠。

**图 4:**
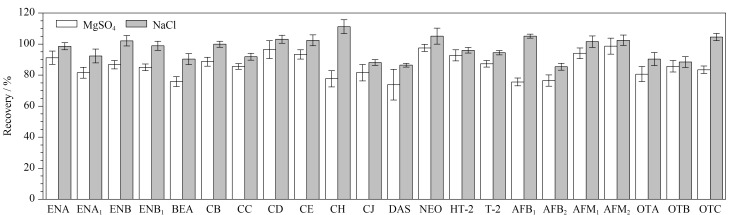
采用不同提取盐时22种真菌毒素的回收率(*n*=6)

### 2.4 基质效应

样品提取净化后,可能会有一些共同提取物对真菌毒素的离子化效率产生影响,造成目标物的信号响应增强或抑制的现象称为基质效应(ME)。基质增强效应或基质抑制效应可能会造成假阳性或者假阴性的结果,因此本文对方法的基质效应做了初步考察。实验选取阴性样品,按照1.3节进行提取净化,获得空白基质提取液。用空白基质提取液和甲醇分别配制混合标准溶液,得到基质匹配校准溶液和溶剂标准溶液,同法分析。

根据ME=(基质匹配校准曲线斜率-溶剂标准曲线斜率)/(溶剂标准曲线斜率)×100%计算。当ME为-20%~20%时为弱基质效应,ME为-50%~-20%或20%~50%时为中等基质效应,当ME≤-50%或≥50%时为强基质效应^[26]^。结果表明,在实验条件下空白基质中22种真菌毒素的基质效应绝对值<20%(见图5),说明牛奶中22种真菌毒素在本实验过程中基质效应较弱。因此本方法采用溶剂标准曲线进行线性回归拟合。

**图5:**
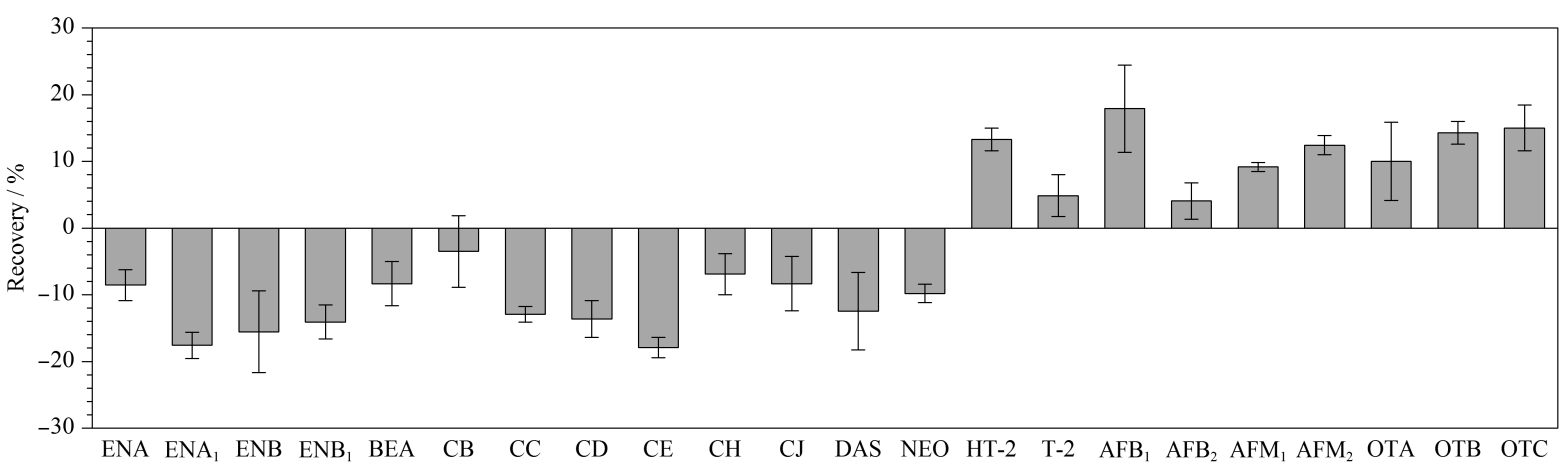
阴性牛奶样品中22种真菌毒素的基质效应(*n*=6)

### 2.5 方法学评价

#### 2.5.1 线性范围与检出限

对不同浓度的标准溶液进行分析,以化合物的峰面积与内标物峰面积之比为纵坐标,质量浓度之比为横坐标,进行线性回归拟合,得到线性方程和相关系数(*R*^2^)。结果表明,22种真菌毒素在0.5~100.0 μg/L范围内线性良好,相关系数均大于0.999。在空白牛奶样品中添加适量标准品,以样品中目标化合物信噪比为3和10时的含量分别为22种真菌毒素的检出限(LOD)和定量限(LOQ),分别为0.3~0.5 μg/kg和1.0~1.5 μg/kg(见表3),低于GB 2761-2017 《食品安全国家标准 食品中真菌毒素限量》^[27]^中规定的指标,说明该方法可满足牛奶中真菌毒素的检测要求。

**表 3:** 22种真菌毒素的线性范围、检出限、定量限及3个加标水平下的回收率和相对标准偏差(*n*=6)

Compound	Linear range/ (μg/L)	Regression equation	LOD/ (μg/kg)	LOQ/ (μg/kg)	Spiked/(μg/kg)	Recoveries/%	RSDs/%
ENA	0.5-100.0	Y=0.7701X-0.456	0.5	1.5	1.5, 5.0, 15	94.7, 94.7, 94.1	4.6, 4.8, 2.2
ENA_1_	0.5-100.0	Y=0.1070X-0.042	0.5	1.5	1.5, 5.0, 15	95.3, 93.9, 93.8	4.9, 3.6, 2.2
ENB	0.5-100.0	Y=0.1136X-0.069	0.5	1.5	1.5, 5.0, 15	97.0, 96.2, 98.3	5.2, 2.8, 3.5
ENB_1_	0.5-100.0	Y=0.1207X-0.063	0.5	1.5	1.5, 5.0, 15	96.7, 98.7, 99.4	6.1, 3.9, 1.2
BEA	0.5-100.0	Y=0.0482X-0.060	0.5	1.5	1.5, 5.0, 15	97.5, 92.3, 97.8	7.9, 5.3, 1.3
CB	0.5-100.0	Y=0.0109X-0.008	0.5	1.5	1.5, 5.0, 15	93.9, 94.1, 93.3	5.3, 5.1, 3.5
CC	0.5-100.0	Y=0.0051X-0.003	0.5	1.5	1.5, 5.0, 15	96.9, 94.2, 95.9	5.1, 4.3, 3.9
CD	0.5-100.0	Y=0.0035X-0.002	0.5	1.5	1.5, 5.0, 15	99.1, 95.4, 97.6	5.3, 4.0, 2.7
CE	0.5-100.0	Y=0.0073X-0.003	0.5	1.5	1.5, 5.0, 15	86.7, 88.6, 89.8	4.1, 3.3, 2.8
CH	0.5-100.0	Y=0.0231X+0.001	0.5	1.5	1.5, 5.0, 15	84.7, 86.5, 86.5	2.4, 1.9, 1.6
CJ	0.5-100.0	Y=0.0090X-0.006	0.5	1.5	1.5, 5.0, 15	85.8, 86.0, 86.7	6.1, 4.3, 3.1
DAS	0.5-100.0	Y=0.0864X-0.015	0.5	1.5	1.5, 5.0, 15	86.2, 87.6, 86.1	8.2, 3.9, 3.7
NEO	0.5-100.0	Y=0.0255X-0.015	0.5	1.5	1.5, 5.0, 15	89.4, 89.5, 89.4	5.6, 3.5, 1.4
HT-2	0.5-100.0	Y=0.0476X-0.012	0.5	1.5	1.5, 5.0, 15	92.3, 92.3, 91.9	3.5, 2.9, 2.3
T-2	0.5-100.0	Y=0.0443X-0.010	0.5	1.5	1.5, 5.0, 15	94.7, 96.7, 95.9	9.9, 3.4, 2.2
AFB_1_	0.5-100.0	Y=0.1029X-0.003	0.3	1.0	1.5, 5.0, 15	100.1, 97.8, 100.2	8.5, 4.4, 1.9
AFB_2_	0.5-100.0	Y=0.0900X-0.004	0.3	1.0	1.5, 5.0, 15	97.8, 96.9, 98.4	4.6, 4.2, 2.2
AFM_1_	0.5-100.0	Y=0.0624X-0.067	0.3	1.0	1.5, 5.0, 15	94.2, 94.7, 94.7	4.5, 2.0, 1.2
AFM_2_	0.5-100.0	Y=0.1121X+0.001	0.3	1.0	1.5, 5.0, 15	94.2, 94.6, 95.2	5.2, 2.5, 1.4
OTA	0.5-100.0	Y=0.0500X-0.064	0.3	1.0	1.5, 5.0, 15	86.4, 87.3, 88.9	3.8, 2.3, 1.2
OTB	0.5-100.0	Y=0.0037X-0.002	0.3	1.0	1.5, 5.0, 15	97.0, 97.0, 100.8	4.6, 3.5, 1.9
OTC	0.5-100.0	Y=0.1566X-0.703	0.3	1.0	1.5, 5.0, 15	89.6, 90.1, 91.9	4.9, 3.4, 1.7

*Y*: peak area ratio of the analyte to isotope internal standard; *X*: mass concentration ratio of the analyte to isotope internal standard.

#### 2.5.2 回收率与精密度

在空白牛奶样品中添加低、中、高3个水平的混合标准工作液,添加量分别为1.5、5.0和15 μg/kg,每个水平做6个平行样。按照优化的前处理条件进行真菌毒素的提取和净化。根据加标浓度和实际测得浓度计算加标回收率和相对标准偏差。结果如表3所示,22种真菌毒素的加标回收率为84.7%~100.8%,相对标准偏差为1.2%~9.9%,说明本实验的重复性和稳定性好,符合要求。

### 2.6 实际样品的检测

购买来自不同国家和地区的市售牛奶25份,用本实验建立的方法检测,实验采用已确证的阴性样品加标,同步进行前处理和分析,作为实验过程的质量控制。实验结果表明,实际样品中均未检出22种真菌毒素。

## 3 结论

本研究建立了牛奶中22种真菌毒素的液相色谱-四极杆/静电场轨道阱质谱快速筛查方法及精准定量检测方法。目标物经酸化乙腈提取后,通过简单的低温高速离心达到净化目的。利用一级全扫描和dd-MS^2^质谱扫描模式,对22种真菌毒素进行了定性定量分析。该方法为开展新兴毒素毒性评价、检测技术研发和污染水平调查提供技术支持,并为提出符合国情的真菌毒素污染控制措施以及食品中新污染物的防控提供依据。
